# Endocrine Crisis Triggered by Pembrolizumab: A Case of Acute-Onset Diabetes With Diabetic Ketoacidosis

**DOI:** 10.7759/cureus.90999

**Published:** 2025-08-25

**Authors:** Riya Raphael, Samraiz Nafees, Aparna Ravikumar, Kailas Nair, Muhammad Haroon Riasat, Eliot Leonard, Shahzad Akbar

**Affiliations:** 1 Internal Medicine, York and Scarborough Teaching Hospitals NHS Foundation Trust, Scarborough, GBR; 2 Accident and Emergency, York and Scarborough Teaching Hospitals NHS Foundation Trust, Scarborough, GBR; 3 Diabetes and Endocrinology, York and Scarborough Teaching Hospitals NHS Foundation Trust, Scarborough, GBR; 4 Internal Medicine, Leeds Teaching Hospitals NHS Trust, Leeds, GBR; 5 Endocrinology and Diabetes, Hull University Teaching Hospitals NHS Trust, Hull, GBR

**Keywords:** diabetic ketoacidosis (dka), endocrinology and diabetes, immuno-oncology, stage iii melanoma, supportive onco-dermatology

## Abstract

Pembrolizumab is a selective monoclonal antibody targeting the programmed cell death protein 1 (PD-1) receptor. It has transformed the landscape of cancer immunotherapy by enabling the immune system to recognise and destroy malignant cells. Despite its efficacy across a range of malignancies, pembrolizumab’s expanding clinical use requires careful consideration of immune-related adverse events (irAEs), including hypopituitarism, adrenocortical dysfunction, thyroid dysfunction, and diabetes mellitus. We report a case of pembrolizumab-induced diabetes mellitus in a previously euglycemic 42-year-old woman treated for stage IIIC melanoma. She developed abrupt-onset, insulin-deficient diabetes presenting as diabetic ketoacidosis, characterized by undetectable C-peptide and negative islet cell autoantibodies, while undergoing adjuvant immunotherapy for stage IIIC melanoma. This report highlights the unpredictable and severe nature of endocrine IRAEs associated with immune checkpoint inhibitors (ICIs), emphasizing the necessity for vigilant monitoring and rapid, multidisciplinary intervention to mitigate morbidity.

## Introduction

Immune checkpoints play a fundamental role in maintaining self-tolerance and regulating immune responses by preventing excessive activation against the body’s own tissues [1]. The surface of immune cells, such as T cells, mediates immune regulation through various receptors, which, upon engagement with specific ligands, deliver inhibitory signals that restrain immune cells from attacking normal tissues [1]. Tumour cells can exploit this mechanism by expressing ligands that bind to these checkpoint-inhibitory receptors, thereby suppressing the immune response [2].

Among the most widely studied inhibitory receptors are cytotoxic T-lymphocyte-associated antigen 4 (CTLA-4) and programmed cell death protein 1 (PD-1) [1,2]. PD-1 is expressed on activated T cells, while its ligand PD-L1 is expressed on tumour cells and antigen-presenting cells. The binding of PD-L1 to PD-1 transmits an inhibitory signal that reduces T-cell proliferation and cytokine production, thereby allowing tumor cells to evade immune surveillance [2].

Since many immune checkpoints are mediated by ligand-receptor interactions, these pathways can be effectively blocked by monoclonal antibodies or modulated using recombinant ligands or receptors [1]. Antibodies directed against CTLA-4, PD-1, and PD-L1, each influencing different stages of T-cell activation, have significantly advanced the field of cancer immunotherapy, particularly in metastatic melanoma [2].

The introduction of agents such as ipilimumab (anti-CTLA-4), nivolumab (anti-PD-1), and pembrolizumab (anti-PD-1) marked a pivotal shift in treatment paradigms, as these therapies block inhibitory immune checkpoints, restore T-cell activity, and have demonstrated improved overall survival in patients with advanced melanoma [2,3]. This therapeutic approach has transformed the management of several malignancies, particularly metastatic and high-risk melanoma, by enhancing antitumour immune responses. However, by overriding immune tolerance mechanisms, immune checkpoint inhibitors (ICIs) also increase the risk of immune-related adverse events (irAEs), which may involve multiple organ systems [4].

Endocrine irAEs are among the most frequently reported toxicities and include hypothyroidism, hyperthyroidism, hypophysitis, primary adrenal insufficiency, and insulin-deficient diabetes mellitus [4,5]. These complications primarily result from cytotoxic T-cell-mediated destruction of endocrine tissues, reflecting a breakdown in immune self-tolerance triggered by checkpoint blockade. One of the rarer but potentially life-threatening endocrine irAEs is ICI-induced insulin-deficient diabetes mellitus with an estimated incidence of 0.2-11% [6]. It often presents acutely and dramatically as diabetic ketoacidosis (DKA), sometimes in patients without prior diabetes or autoimmune disease. Early recognition is critical, as delayed diagnosis can cause significant morbidity and may require interruption or cessation of immunotherapy.

In this report, we present a case of new-onset insulin-dependent diabetes manifesting as DKA shortly after initiation of pembrolizumab. This case highlights the importance of maintaining a high index of suspicion for endocrine complications during ICI therapy and underscores the need for clear screening and management guidelines to facilitate timely diagnosis and minimise treatment interruptions.

## Case presentation

A 42-year-old female with a history of stage IIIC melanoma was undergoing adjuvant therapy with pembrolizumab. She presented to the emergency department three weeks after her third infusion of pembrolizumab, reporting a five-day history of progressively worsening polydipsia, abdominal discomfort, and dyspnoea. Her medical history was significant for hypothyroidism, secondary to previous treatment for hyperthyroidism, for which she was taking levothyroxine, with thyroid function well controlled prior to initiating pembrolizumab, as documented in her records. She also had a history of hypertension and no prior history of diabetes mellitus.

On presentation, laboratory investigations revealed marked hyperglycemia, severe metabolic acidosis, elevated serum ketones, and a raised lactate level, as described in Table 1. There was no clinical or laboratory evidence of infection or significant electrolyte derangement. These findings were consistent with a diagnosis of DKA.

**Table 1:** Laboratory investigations at the time of presentation

Arterial blood gases	Value	Normal range
pH	7.10	7.35-7.45
Bicarbonate	9 mmol/L	22-28 mmol/L
Serum glucose	26.2 mmol/L	4.0 - 7.0 mmol/L
Serum ketones	6	<0.6
Lactate	4.5 mmol/L	0.5 - 2.2 mmol/L
Serum sodium	139 mmol/L	135-145 mmol/L
Serum potassium	4.1 mmol/L	3.5-5.0 mmol/L
CRP	11 mg/L	0-5 mg/L

Further laboratory assessment showed a rapid rise in HbA1c from 39 mmol/mol (measured four weeks earlier) to 69 mmol/mol at presentation. Endocrine workup demonstrated undetectable C-peptide levels, as detailed in Table 2, consistent with profound insulin deficiency, with negative results for islet autoantibodies (anti-GAD, IA2, and ZnT8). This finding effectively rules out classical autoimmune type 1 diabetes and is instead suggestive of pembrolizumab-mediated, immune-related β-cell dysfunction.

**Table 2:** Endocrine investigations

Laboratory investigations	Value	Normal range
HbA1C at the time of presentation	69 mmol/mol	<42 mmol/mol
C-peptide	<7 pmol/L	100-300 pmol/L (random/postprandial)

Given the temporal proximity to pembrolizumab administration and the absence of alternative precipitating factors or newly introduced medications, the ICI was identified as the most likely etiological factor for the development of acute-onset insulin-deficient diabetes. The patient was discharged on a subcutaneous basal-bolus insulin regimen with instructions for regular monitoring of capillary blood glucose levels at home.

Follow-up was arranged with Endocrinology for the optimisation of the insulin regimen and further evaluation, including assessment of pituitary and adrenal function post-discharge to monitor for additional immune-related endocrinopathies; with the Diabetes Specialist Team to provide structured education and ongoing support in insulin management; and with Oncology services to evaluate the risk-benefit profile of continuing pembrolizumab therapy and to guide subsequent management strategies for melanoma.

## Discussion

DKA is a rare but potentially life-threatening IRAE associated with immune checkpoint inhibitors (ICIs), including pembrolizumab. Although the incidence of ICI-induced DKA is low - estimated at approximately 0.1% in clinical trials - its consequences can be severe if not promptly recognised and managed [7,8]. This condition typically resembles type 1 diabetes mellitus, characterised by its abrupt onset and an immediate requirement for insulin therapy. Notably, most patients do not exhibit diabetes-related autoantibodies and often lack a prior history of diabetes [6].

The proposed mechanism underlying the development of diabetes in patients undergoing ICI therapy involves activation of autoreactive T cells secondary to PD-1 inhibition [9]. Pembrolizumab blocks PD-1 receptors, preventing interaction with PD-L1 molecules expressed on pancreatic β cells. This blockade results in unchecked autoreactive CD8+ T-cell-mediated destruction of pancreatic islet cells, leading to insulin deficiency and the onset of type 1 diabetes mellitus [9]. In some cases, as observed in this report, pembrolizumab may precipitate acute deterioration of preexisting prediabetes or type 2 diabetes mellitus [9,10].

Current clinical guidelines do not recommend routine glucose monitoring for all patients receiving ICIs; however, accumulating case reports highlight the importance of vigilant monitoring. These data underscore the need for routine assessment of thyroid function and blood glucose levels both prior to and throughout ICI therapy to facilitate early detection and intervention [9,11].

## Conclusions

This case highlights the importance of recognising pembrolizumab-induced diabetes as a potentially life-threatening, immune-related adverse event. Timely diagnosis, vigilant symptom monitoring, and proactive screening, supported by close collaboration between oncology and endocrinology teams, are essential to optimise patient outcomes and ensure the safe continuation of immunotherapy. Given the current lack of standardised national guidelines for routine endocrine monitoring during ICI therapy, particularly in relation to blood glucose levels and endocrine dysfunction, reporting such cases is vital. These reports not only raise clinical awareness but also contribute to the development of evidence-based practices for the early identification and management of serious endocrine toxicities associated with immunotherapy.

## References

[1] Pardoll DM (2012). The blockade of immune checkpoints in cancer immunotherapy. Nat Rev Cancer.

[2] Shah V, Panchal V, Shah A, Vyas B, Agrawal S, Bharadwaj S (2024). Immune checkpoint inhibitors in metastatic melanoma therapy (Review). Med Int (Lond).

[3] Zand Irani A, Almuwais A, Gibbons H (2022). Immune checkpoint inhibitor-induced diabetes mellitus with pembrolizumab. BMJ Case Rep.

[4] Barroso-Sousa R, Barry WT, Garrido-Castro AC, Hodi FS, Min L, Krop IE, Tolaney SM (2018). Incidence of endocrine dysfunction following the use of different immune checkpoint inhibitor regimens: a systematic review and meta-analysis. JAMA Oncol.

[5] Byun DJ, Wolchok JD, Rosenberg LM, Girotra M (2017). Cancer immunotherapy - immune checkpoint blockade and associated endocrinopathies. Nat Rev Endocrinol.

[6] Stamatouli AM, Quandt Z, Perdigoto AL (2018). Collateral damage: insulin-dependent diabetes induced with checkpoint inhibitors. Diabetes.

[7] Pacichana JA, Osorio LM, Restrepo K, García AF, Rivas G, Liscano Y (2024). Diabetic ketoacidosis as a debut and immune-mediated complication caused by pembrolizumab: case report. Diabetology.

[8] Salangsang J, Sapkota S, Kharel S, Gupta P, Kalla A (2023). A case of pembrolizumab-induced diabetic ketoacidosis and hyperthyroidism in a patient with recurrent esophageal adenocarcinoma. Cureus.

[9] Clotman K, Janssens K, Specenier P, Weets I, De Block CE (2018). Programmed cell death-1 inhibitor-induced type 1 diabetes mellitus. J Clin Endocrinol Metab.

[10] Kichloo A, Albosta MS, McMahon S (2020). Pembrolizumab-induced diabetes mellitus presenting as diabetic ketoacidosis in a patient with metastatic colonic adenocarcinoma. J Investig Med High Impact Case Rep.

[11] Kotwal A, Perlman JE, Goldner WS, Marr A, Mammen JS (2023). Endocrine dysfunction from immune checkpoint inhibitors: pearls and pitfalls in evaluation and management. JCO Oncol Pract.

